# Effects of tangerine peel flavonoids on flavor compounds and microbial communities in cigar tobacco fermentation: insights from ion migration chromatography and microbiomics

**DOI:** 10.3389/fmicb.2026.1793725

**Published:** 2026-05-04

**Authors:** Jian Zhang, Yinfeng Ren, Yun Jia, Bo Li, Chong Chen, Wanrong Hu, Shiru Jia, Dongliang Li, Yuanfa Liu

**Affiliations:** 1 State Key Laboratory of Food Nutrition and Safety, Laboratory of Industrial Fermentation Microbiology (Ministry of Education),College of Biotechnology, Tianjin University of Science and Technology, Tianjin, China; 2 Future Food (Baima) Research Institute, Nanjing, China; 3 China Tobacco Technology Innovation Center for Cigar, China Tobacco Sichuan Industrial Co., Ltd., Chengdu, China; 4 School of Grain Science and Technology, Jiangsu University of Science and Technology, Zhenjiang, China; 5 State Key Laboratory of Food Science and Technology, School of Food Science and Technology, National Engineering Research Center for Functional Food, National Engineering Laboratory for Cereal Fermentation Technology, Collaborative Innovation Center of Food Safety and Quality Control in Jiangsu Province, Jiangnan University, Wuxi, China

**Keywords:** cigar tobacco leaves, fermentation technology, flavor, microbial communities, tangerine peel flavonoids

## Abstract

**Introduction:**

It is reported that addition of tangerine peel extract (flavonoids) can significantly improve the quality and flavor of tobacco leaves during fermentation. Nobiletin, tangeretin and hesperidin are the predominant flavonoids present in tangerine peel. However, their effects on the fermentation process of cigar tobacco leaves (CTL) have not yet been elucidated.

**Methods:**

Three major tangerine peel flavonoids, hesperidin (CPG), nobiletin (CCPS) and tangeretin (JPS), were applied during the fermentation of CTL) to evaluate their effects on fermentation outcomes. Gas chromatography–ion mobility spectrometry (GC-IMS) and microbiome analysis based on 16S rRNA and internal transcribed spacer sequencing were employed to characterize volatile flavor compounds and microbial community dynamics during the 30 days of fermentation.

**Results:**

A total of 157 volatile compounds were identified in CTL in this study. All tangerine peel flavonoid treatments improved the flavor quality of tobacco leaves by modulating the microbial community. The CPG group was enriched in esters and acids (e.g., n-amyl formate and propyl acetate), contributing to fruity aroma, with high relative abundances of *Firmicutes* and *Staphylococcus*, and a slight increase in *Bacillus*. The CCPS group accumulated abundant ketones (e.g., 3-methyl-2-cyclopenten-1-one, 3-pentanone, and 2-hydroxy-2-methyl-4-pentanone), providing caramel-like sweetness and enhancing the smoke body, while the enrichment of *Sphingomonas*, *Rhizobium* and other genera elevated bacterial diversity. The JPS group formed characteristic volatile compounds showed the highest fungal richness among all treatments. The mixed flavonoid (HHHT) group exhibited the highest abundance of *Firmicutes* and *Staphylococcus*, the lowest proportion of *Proteobacteria*, nearly undetectable harmful microorganisms, and the lowest microbial diversity. All treatment groups shared certain microbial trends, but distinct microbial profiles were observed among the CPG, CCPS, JPS, and HHHT groups, with significant correlations between key microorganisms and volatile flavor compounds

**Discussion:**

In this study, the influence of tangerine peel flavonoids on CTL flavor quality and microbial community was systematically analyzed. These findings demonstrated that tangerine peel flavonoids enhanced flavor of CTL by modulating microbial communities during fermentation, and this provides new insights into CTL processing.

## 1 Introduction

Cigars have gained widespread acceptance among consumers. Unlike many traditional tobacco products, cigars are processed through the curing, fermentation, rolling, and aging. These processes bring in rich smoky flavors, mellow aromas, and distinctive sensory characteristics (Zhang M. Z. et al., 2024). However, compared with traditional Chinese flue-cured tobacco, research on Chinese cigar tobacco was initiated relatively late. In addition, compared with high-quality foreign cigar tobaccos, Chinese cigar tobaccos generally exhibit less distinctive fewer distinctive flavor characteristics, and this results in weaker market competitiveness.

Cigar tobacco leaf (CTL) quality can be enhanced by well-designed fermentation regimens. Such regimens help to reduce inherent defects. They also alter the chemical composition. Furthermore, they boost tobacco-specific aroma. Accordingly, the quality of CTL can be further enhanced through the incorporation of fermentation additives. The fermentation additives used in cigar production commonly include microbial agents, enzyme preparations and natural extracts (Hu et al., 2022; Wu et al., 2023). Natural extract-based fermentation medium is generally classified as tobacco-derived or non-tobacco-derived, and the non-tobacco-derived fermentation additives are typically extracted from edible plant materials. For instance, coffee and cocoa extracts can enhance the richness, mellowness, and maturity of cigar aroma, as well as the smoke’s smoothness and sweetness (Hu et al., 2023; Zong et al., 2023). Similarly, ginger extracts and “Humi” (coking rice–water paste) have been reported to enhance aroma and mellowness while reducing off-flavor formation (Hu et al., 2022; Ren et al., 2024). The addition of plant extracts brings inherent characteristic flavor, and it also affects the activity of microorganisms in CTL. The synergistic effect ultimately improves the quality of cigars (Wen et al., 2024).

As important plant-derived bioactive components, flavonoids not only exhibit strong antioxidants and antimicrobial activities, but also serve as potential regulators of microbial growth and enzyme activity. Recent studies have confirmed that the addition of flavonoids during the fermentation of cigar tobacco leaves can effectively regulate the structure and function of microbial communities, promote the degradation of harmful substances, and enhance the accumulation of aroma compounds. This further reduces irritation, improves flavor quality, and ultimately enhances the overall quality of cigar tobacco leaves (Hu et al., 2024).

Tangerine peel, the dried mature pericarp of Citrus reticulata Blanco, is predominantly cultivated in several regions of China (Qin et al., 2024). It has been used medicinally for centuries and has been reported to exert effects such as regulating Qi, invigorating the spleen, drying dampness, and resolving phlegm (Yi et al., 2015; Fu et al., 2017). Hesperidin (CPG), nobiletin (CCPS), and tangeretin (JPS) are the major flavonoids in tangerine peel, with diverse biological activities including anti-inflammatory, anti-tumor, and antifungal properties. Fermentation of CTL is a complex biochemical process co-mediated by endogenous enzymes and microbial communities, which directly determines the aroma, flavor, and quality of tobacco (Zhang et al., 2025). However, the specific flavonoid monomers influencing the fermentation process of CTL remain unclear. Hesperidin (CPG), nobiletin (CCPS), and tangeretin (JPS) are the major flavonoids in tangerine peel and exhibit diverse biological activities, including anti-inflammatory, anti-tumor, and antifungal properties. CPG, CCPS, and JPS may act as substrates or antimicrobial agents, thereby differentially affecting the structure and function of microbial communities during fermentation. The sugar moieties released by hydrolysis of flavonoid glycosides may provide carbon sources for microorganisms, and flavonoids may also serve as signaling molecules to regulate microbial gene expression and metabolism. Some flavonoids may exert antimicrobial activity and selectively inhibit harmful microorganisms, thereby optimizing the fermentation process. In addition, flavonoids may modulate the activities of microbial enzymes, thereby affecting the degradation and transformation of macromolecules such as proteins and carbohydrates in tobacco leaves, and ultimately influencing the overall fermentation process and metabolite formation of CTL (Zhang L.-Y. et al., 2024).

In this study, three major flavonoid monomers from tangerine peel, namely CPG, CCPS, and JPS, were selected as research objects and were added individually or as an equimolar mixture to the CTL fermentation system. Integrating headspace-gas chromatography-ion mobility spectrometry (HS-GC-IMS) with high-throughput sequencing of 16S rRNA and internal transcribed spacer (ITS) genes, we elucidated the effects of different tangerine peel flavonoid treatments on the composition of volatile flavor compounds and the structure of microbial communities during CTL fermentation. We further identified the regulatory effects of key flavonoid monomers on CTL fermentation, identified the core microbial taxa associated with flavor formation, and explored the correlation between microbial communities and flavor compounds. This study aims to provide a theoretical basis and practical reference for the directional optimization of CTL fermentation technology and the improvement of flavor quality of domestic CTL via the addition of tangerine peel flavonoids.

## 2 Materials and methods

### 2.1 Plant materials

CTL (Dexue No. 1) were obtained from Sichuan China Tobacco Industry Co., Ltd.

Xinhui Chenpi aged for 5 years, supplied by Guangdong Yixiang Chenpi Co., Ltd. It is cryogenically ground with liquid nitrogen and then sieved through an 80-mesh sieve for reserve.

### 2.2 Equipment and reagents

FlavourSpec^®^ Gas Chromatography-Ion Mobility Spectrometer (GC-IMS), G.A.S. Company (Dortmund, Germany); CTC-PAL 3 Static Headspace Autosampler, CTC Analytics AG (Zwingen, Switzerland); GC-2010Pro Gas Chromatograph (GC), Shimadzu Corporation (Kyoto, Japan); SCIEX 5500 Liquid Chromatography-Triple Quadrupole Mass Spectrometer (LC-MS/MS), SCIEX (Framingham, MA, United States); LHS-250HC-I Constant Temperature and Humidity Incubator, Shanghai Yiheng Scientific Instrument Co., Ltd. (Shanghai, China); GCMS-TQ8040 NX Triple Quadrupole Gas Chromatography-Mass Spectrometer (GC-MS/MS), Shimadzu Corporation (Kyoto, Japan); 2720 PCR Amplifier, Applied Biosystems (ABI, Foster City, CA, United States); DYY-6C Electrophoresis Apparatus, Beijing Liuyi Biotechnology Co., Ltd. (Beijing, China); BG-gdsAUTO (130) Gel Imaging System, Beijing Baijing Biotechnology Co., Ltd. (Beijing, China).

### 2.3 Sample preparation

Flavonoids (CPG, JPS, and CCPS) were extracted from dried tangerine peels according to the method described by Wang et al. (2005), and their purity was confirmed to be 98%. The moisture content of the CTL was first determined. The required volume of rehydration water to adjust the leaf moisture content to 28% was calculated using a weighing method. After moisture equilibrium was achieved, the leaves were placed in a temperature- and humidity-controlled incubator (35°C, 75% relative humidity) for fermentation. For the flavonoid treatment groups, CPG, CCPS, JPS, and their equimolar mixture (HHHT) were added to the tobacco leaves at a concentration of 3‰ (w/w). The calculated volume of rehydration water was used to dissolve the flavonoid components, and the resulting solution was uniformly sprayed onto the tobacco leaves. Fermentation was conducted for 30 days. After fermentation, the samples were immediately ground in liquid nitrogen and stored at −80°C. The blank control group was fermented with sterile water, whereas samples from the unfermented group and the fermented control group were collected at 0 and 30 days, respectively.

### 2.4 HS-GC-IMS analysis

The volatile compounds of CTL was identified according to the method of Feng et al. (2022) with some modification. Briefly, 0.5 g of each sample was weighed into a 20 mL headspace vial, followed by the addition of 2 mL of pure water, 0.1 g of aspartic acid, and 50 μL of 100 ppm 2-octanol as the internal standard. The vial was then incubated at 90°C for 15 min prior to injection. The GC program parameters were as follows: incubation temperature, 90°C; incubation time, 15 min; injection volume, 500 μL; injection mode, split less; incubation speed, 500 rpm; and injection needle temperature, 110°C. The column temperature was maintained at 80°C, and high-purity nitrogen (≥ 99.999%) was used as the carrier gas. The gas flow rate program was as follows: an initial flow rate of 2.0 mL/min maintained for 5 min, followed by a linear increase to 10.0 mL/min over 10 min, a further increase to 100.0 mL/min over 15 min, and a final hold for 20 min. The total chromatographic run time was 50 min, and the injection port temperature was set at 80°C. IMS parameters were as follows: ionization source, tritium (^3^H); drift tube length, 53 mm; electric field strength, 500 V/cm; drift tube temperature, 45°C; drift gas, high-purity nitrogen (≥ 99.999%) at a flow rate of 150.0 mL/min; ion mode, positive; gate open time, 100 μs; and gate voltage, 90 dgt.

### 2.5 Sensory quality evaluation

CTL fermented with different flavonoids (including the control group) were hand-rolled into cigars. Their sensory quality—specifically aroma, smoke characteristics, taste, and combustion properties—was evaluated by a panel of five experts from Sichuan China Tobacco, following the tobacco industry standard YC/T415—2011 Sensory Evaluation Methods for Tobacco Products.

### 2.6 Microbial community analysis

Total microbial DNA was extracted using the OMEGA Soil DNA Kit (D5625-01, Omega Bio-Tek, Norcross, GA, United States), and the DNA concentration was determined using a UV–visible spectrophotometer. The hypervariable V3–V4 region of the bacterial 16S rRNA gene (∼468 bp) was selected for sequencing and amplified by PCR using the primer pair 338F (5′-barcode + ACTCCTACGGGAGGCAGCA-3′) and 806R (5′-GGACTACHVGGGTWTCTAAT-3′). PCR amplification of fungal ITS was performed using the primers F: CTTGGTCATTTAGAGGAAGTAA and R: GCTGCGTTCTTCATCGATGC. Sequencing libraries were constructed using the TruSeq Nano DNA LT Library Prep Kit (Illumina), followed by paired-end sequencing (2 × 250 bp) on an Illumina Nova Seq platform using the NovaSeq 6000 SP Reagent Kit (500 cycles).

### 2.7 Statistical analysis

Data analysis was performed using SPSS 26.0 and Microsoft Excel. Graphical representations were generated using Origin 2024. Partial least squares–discriminant analysis (PLS-DA) was conducted using SIMCA 14.1. Principal component analysis (PCA), hierarchical clustering, and heatmap construction were performed using R packages and TBtools. Microbial community data were analyzed using the GenesCloud online platform.^1^ All experiments were conducted with three biological replicates. Target compounds were qualitatively identified by searching and matching against the built-in GC retention index database (NIST) and IMS drift time database in the VOCal software, and plugins such as Reporter and Gallery Plot in the same software were used to generate two-dimensional spectra, difference spectra and fingerprint spectra of volatile components for the comparative analysis of volatile organic compounds among different.

## 3 Results and discussion

### 3.1 Flavor profile of CTL by GC-IMS

#### 3.1.1 Analysis of volatile component types and contents

**FIGURE 1:**
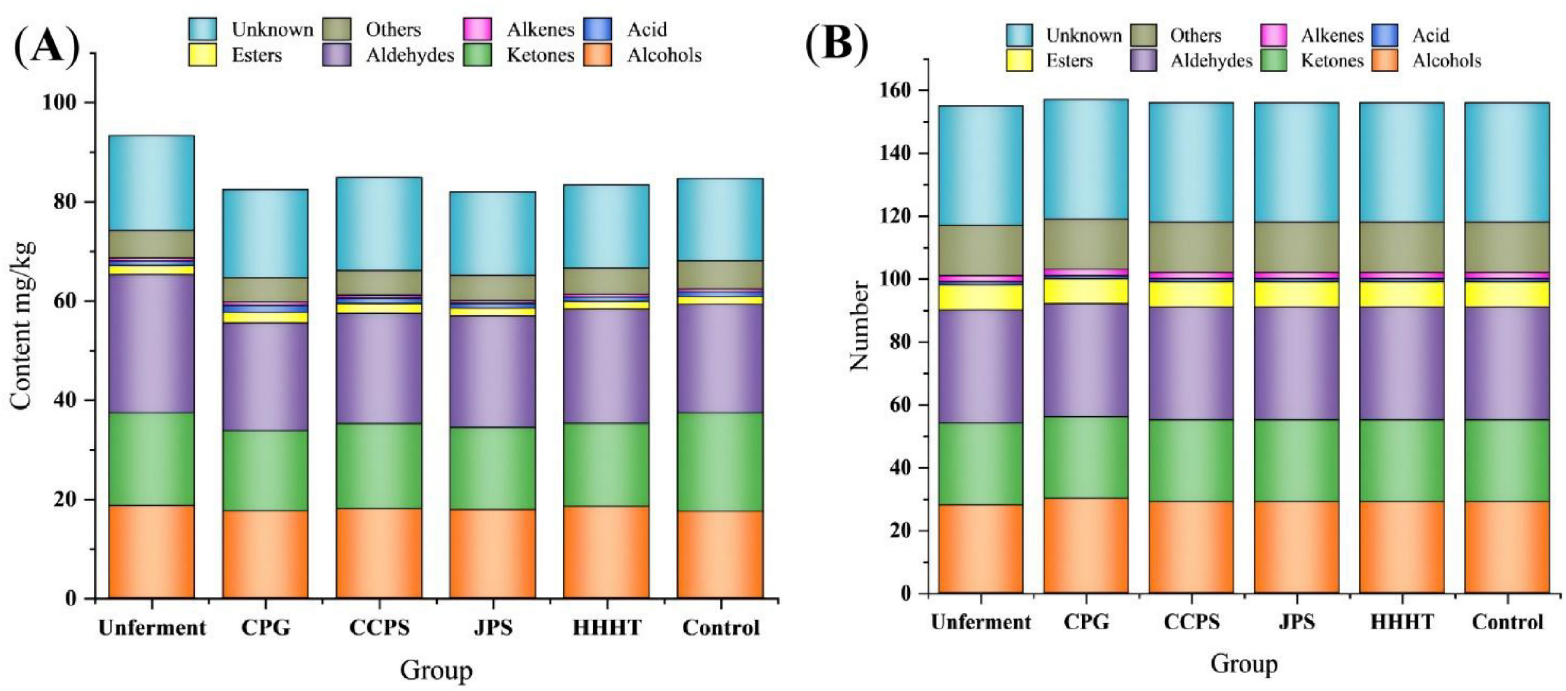
Flavor substances in different fermentation groups. **(A)** Substance content. **(B)** Substance types.

A total of 157 volatile compounds were detected via HS-GC-IMS. Qualitative identification was conducted by comparing the drift and retention times of the detected compounds with those in the NIST and IMS databases integrated into the HS-GC-IMS system, and 119 volatile compounds (including dimers and trimers) were identified (Raza et al., 2020). As shown in Figures 1A,B, the 157 volatile compounds were classified into 30 alcohols, 26 ketones, 36 aldehydes, 8 esters, 2 alkenes, 1 acid, and 38 unidentified compounds. The unfermented group exhibited a higher total volatile content than the fermented groups. This difference may be associated with microbial metabolism during fermentation, during which certain volatile components were converted into smaller molecules, leading to a reduction in overall volatile content (Zhang et al., 2025). Among all fermentation groups, the CPG group exhibited the lowest levels of volatile compounds (i.e., alcohols, ketones, and aldehydes) after 30 days of fermentation with different tangerine peel flavonoid media. The corresponding concentrations were 17.501 ± 0.074 mg/kg, 16.182 ± 0.142 mg/kg, and 21.814 ± 0.416 mg/kg, respectively. In contrast, the CPG group exhibited the highest levels of esters and acids, with concentrations of 2.112 ± 0.062 mg/kg and 1.456 ± 0.143 mg/kg, respectively. The CCPS fermentation group exhibited the highest ketone content among all fermentation groups, with a concentration of 17.193 ± 0.216 mg/kg.

#### 3.1.2 Volatile compound profiling and group differences

**FIGURE 2:**
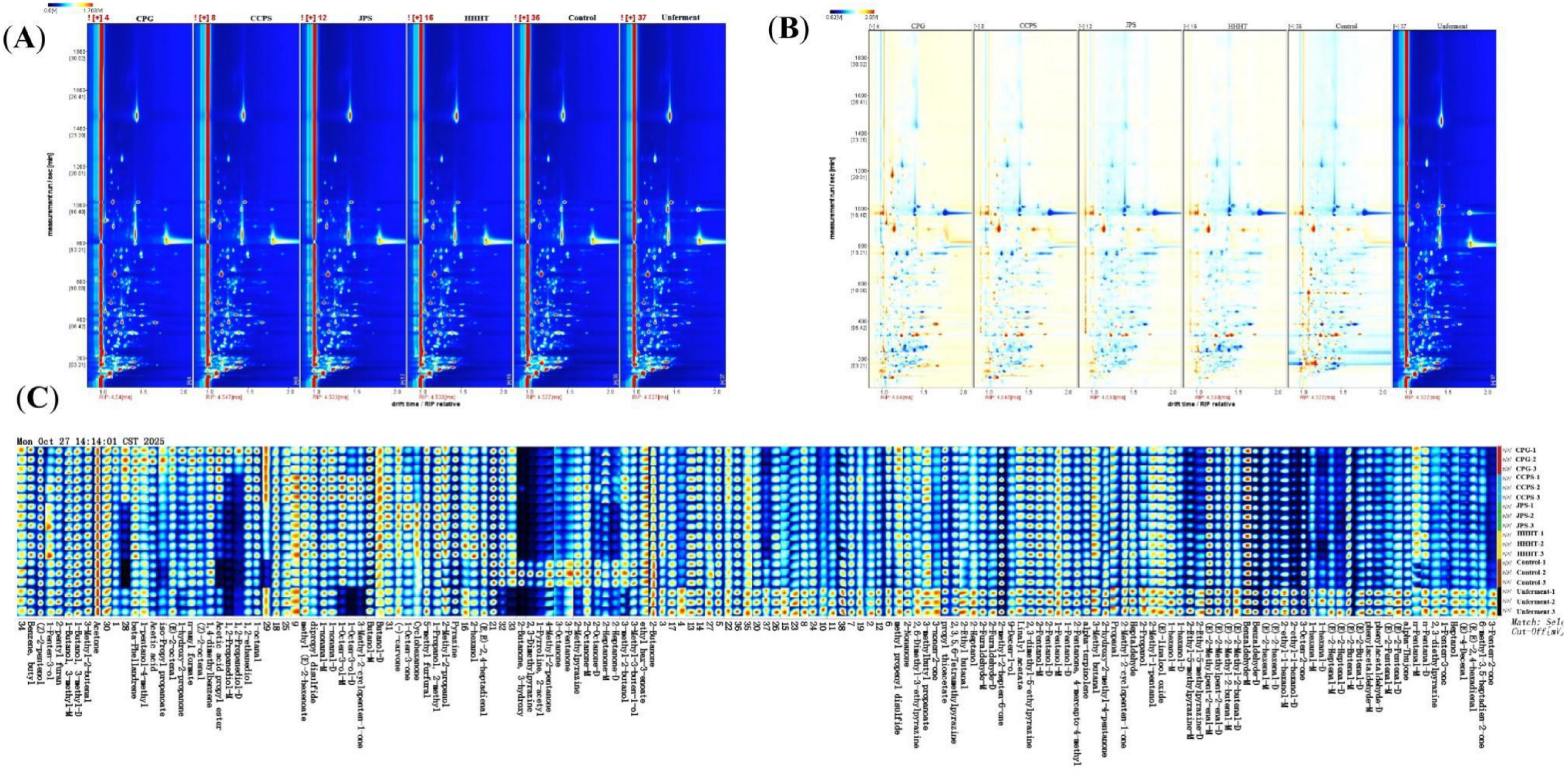
Analysis of volatile organic compounds (VOCs) in different fermentation groups. **(A)** GC-IMS two-dimensional (2D) spectra. **(B)** GC-IMS difference spectra (reference: CPG group; red = higher content, blue = lower content). **(C)** VOC fingerprint profiles.

Figure 2 illustrates the differences in volatile components among the different fermentation groups. In the two-dimensional spectrum (Figures 2A,B), volatile components in tobacco leaf samples were separated by GC within 1,200 s and by IMS within 2 ms. The positions and intensities of ion signals were relatively consistent across samples, with differences mainly observed in color intensity, indicating that the types of volatile components were largely conserved whereas their concentrations varied. For comparative analysis, the spectrum of the unfermented group was used as the reference. Differential comparison profiles for each group were generated by subtracting the spectra of the other fermentation groups from hose of the reference group.

The complete VOC profiles of tobacco leaf samples from different fermentation groups, along with the differences among samples, are presented in Figure 2C. Compared with the reference group, the CPG fermentation group exhibited elevated levels of acids and esters, represented by acetic acid, n-amyl formate, and propyl acetate. Similarly, increased contents of 1-non-anal, 1-octen-3-ol, and 3-methyl-2-cyclopenten-1-one were observed in the CCPS fermentation group. The JPS fermentation group showed elevated levels of (-)-carvone, 1-octen-3-one, and cyclohexanone. Meanwhile, enhanced concentrations of 2-hexanol and (E,E)-2,4-heptadienal were detected in the HHHT fermentation group. These results demonstrated that different flavonoid-modulated fermentation treatments led to distinct alterations in the profiles of flavor-related volatile compounds.

**FIGURE 3:**
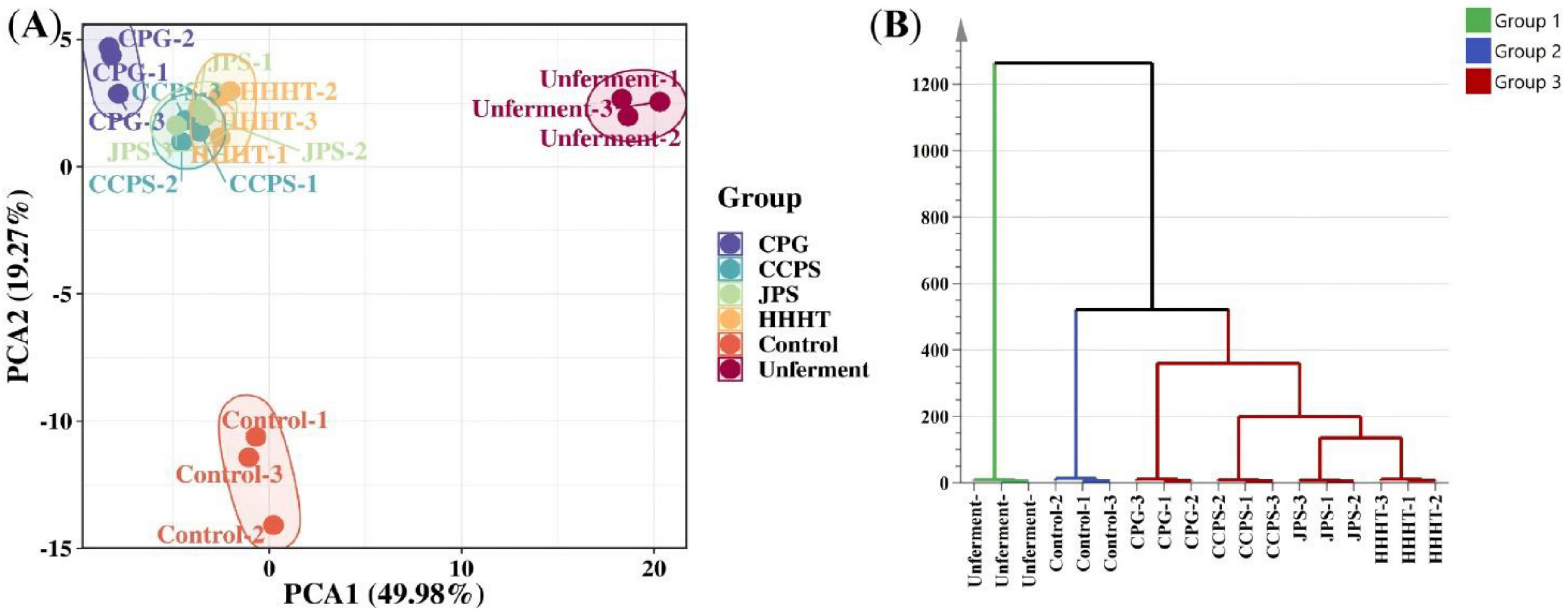
Analysis of samples from different fermentation groups. **(A)** PCA score plot. **(B)** HCA results.

**FIGURE 4:**
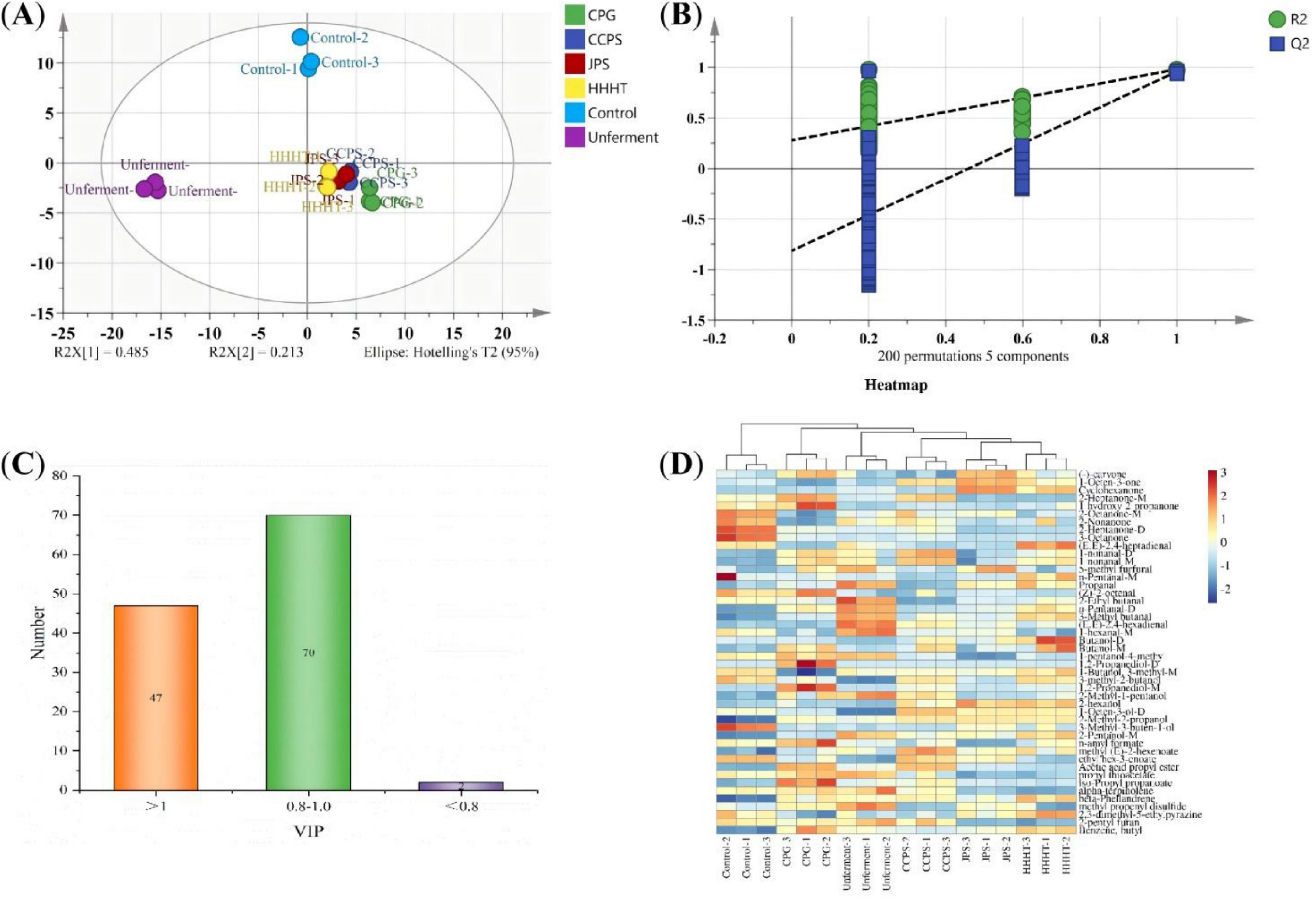
OPLS-DA analysis of tobacco leaf samples from different fermentation groups. **(A)** OPLS-DA score plot. **(B)** Model permutation test results (200 times). **(C)** Number of substances with different VIP values. **(D)** Heatmap of differential substances with VIP > 1 in different groups.

The results of PCA and hierarchical clustering analysis (HCA) for tobacco leaf samples from different fermentation groups are shown in Figure 3. The first principal component (PC1) and the second principal component (PC2) explained 49.98% and 19.27% of the total variance, respectively, accounting for a cumulative variance of 69.25% (Figure 3A). This result indicates that most of the variance in the original dataset was retained. Samples from different groups were effectively clustered, with the control and unfermented groups clearly separated from those treated with tangerine peel flavonoid media. Figure 3B shows that HCA and PCA yielded consistent results, confirming significant differences between tobacco leaf samples treated with tangerine peel flavonoid media and the control and unfermented groups. These results further demonstrate the suitability of HS-GC-IMS for the analysis of volatile components in tobacco leaves. Orthogonal Partial Least Squares–Discriminant Analysis (OPLS-DA) was employed to model the volatile components. The OPLS-DA results, permutation test outcomes, and variable importance in projection (VIP) values for volatile components in tobacco leaf samples from different fermentation groups are shown in Figure 4. Figure 4A shows that the OPLS-DA model effectively distinguished the control, unfermented, and flavonoid-treated groups (CPG, CCPS, JPS, and HHHT), with R^2^X = 0.938, R^2^Y = 0.976, and Q^2^ = 0.942. These favorable R^2^ and Q^2^ values indicate good model fitting, stable and reliable predictive performance, and acceptable interpretability (Chang et al., 2025). Results from 200 cross-validation permutation tests showed that the predicted Q^2^ values intersected the negative half of the Y-axis, suggesting that the model achieved good predictive performance without overfitting (Yan et al., 2025). These results support the applicability of the OPLS-DA model for identifying differences among tobacco leaves from different fermentation groups.

**FIGURE 5:**
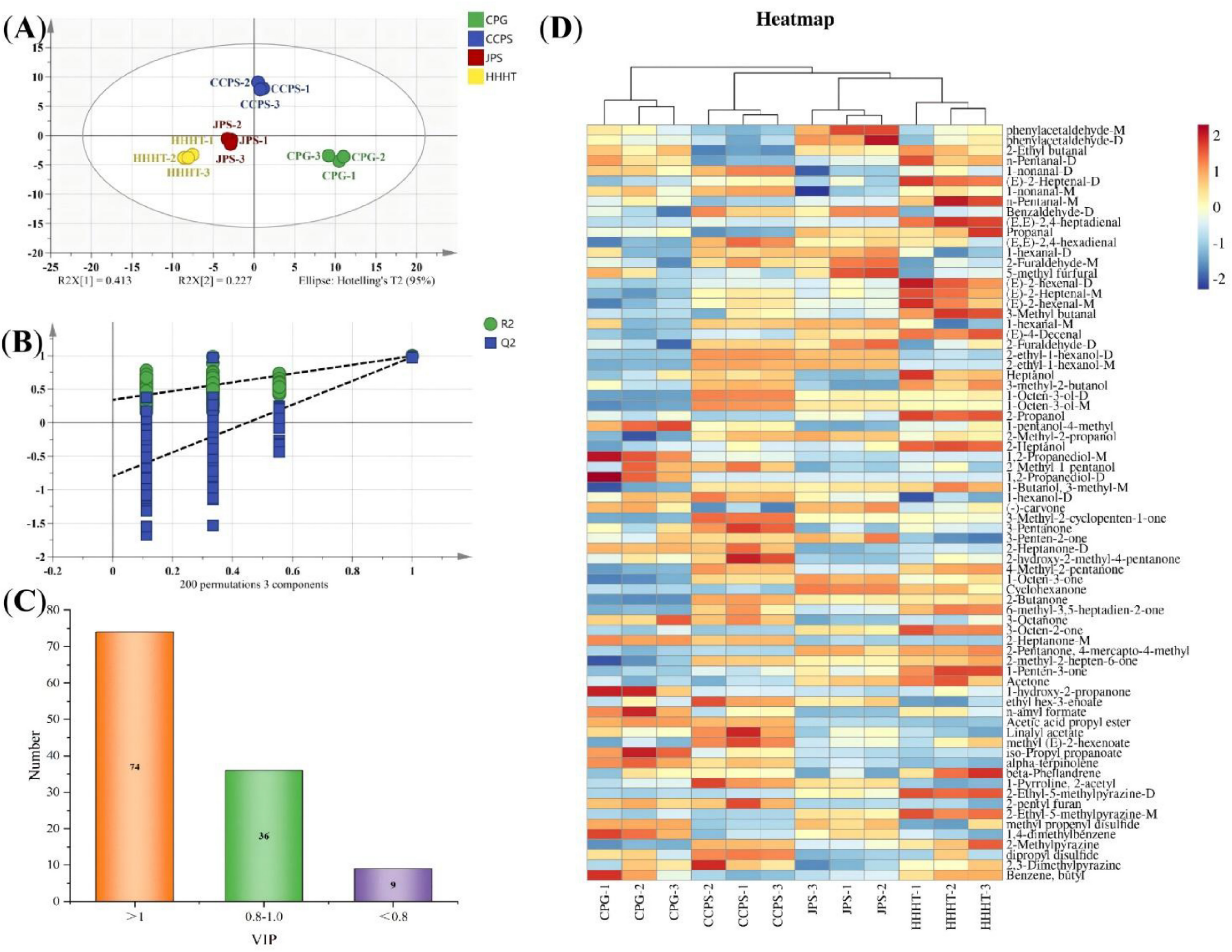
OPLS-DA analysis of CPG, CCPS, JPS, and HHHT groups. **(A)** OPLS-DA score plot. **(B)** Model permutation test results. **(C)** VIP plot. **(D)** Heatmap of differential substances with VIP > 1.

Based on the criterion of VIP > 1, a total of 47 differential volatile components were identified (Supplementary Table S1). A heatmap of these differential volatile components across fermentation groups is shown in Figure 4D, showing significant differences in the contents of the 47 compounds among groups (*p* < 0.05). Among these compounds, 2-octanone-M, 2-nonanone, and 3-methyl-3-buten-1-ol were most abundant in the control group, whereas 2-ethylbutanal, 3-methylbutanal, and (E,E)-2,4-hexadienal were most abundant in the unfermented group. CTL samples treated with tangerine peel flavonoid fermentation showed significant differences compared with the control and unfermented groups (Figure 4A). OPLS-DA was performed to examine differences in volatile compounds among the flavonoid-treated groups (CPG, CCPS, JPS, and HHHT), and the results are shown in Figure 5. The OPLS-DA model effectively distinguished the four flavonoid-treated groups, with R^2^X = 0.812, R^2^Y = 0.987, and Q^2^ = 0.963 (Figures 5A,B).

These parameters further support the robustness of the OPLS-DA model. Results from 200 cross-validation permutation tests confirmed that the model exhibited no overfitting and achieved good predictive performance. As shown in Figure 5C, a total of 74 volatile compounds with VIP > 1 were identified (Supplementary Table S2). A heatmap of these differential compounds is presented in Figure 5D, showing clear intergroup differences. In the CPG group, representative enriched compounds included 4-methyl-1-pentanol, n-amyl formate, and isopropyl propanoate. The CCPS group showed higher levels of 1-nonanal, 1-octen-3-ol, and 3-methyl-2-cyclopenten-1-one. The JPS group was characterized by phenylacetaldehyde, (-)-carvone, and 1-octen-3-one. The HHHT group showed elevated levels of 2-ethylbutanal, (E,E)-2,4-heptadienal, and acetone.

### 3.2 Sensory evaluation analysis

Sensory evaluation of the fermented cigar tobacco leaves provided an intuitive reflection of the changes in their flavor quality. As shown in Table 1, compared with the control group, the sensory quality of all flavonoid fermentation groups improved to varying degrees, confirming the feasibility of using tangerine peel flavonoids as a medium for fermenting cigar tobacco leaves.

**TABLE 1:** Sensory evaluation scores of cgar tobacco leaves fermented with different flavonoids

Sensory indicators		The scoring scale ranged from 0 to 9, where 0–3 indicated poor quality, 3–6 indicated moderate quality, and 6–9 indicated good quality.				
		CPG	CCPS	JPS	HHHT	Control
Aroma	Aroma quantity	5	6	5	5	4.5
	Richness	5	6	5	5	4.5
	Maturity	5.5	5	5.5	5	4.5
Smoke characteristics	Irritancy	5	5	5	5	4.5
	Mellowness	4.5	5	5	4.5	4.5
	Fineness	5	6.5	5	5	5
Taste	Sweetness	5	6	5	5	5
	Cleanness	4.5	6	5	5	4
	Aftertaste	4.5	5	4.5	4.5	4.5
Combustion properties	Combustion	7	7	6.5	6	6
	Ash color	6	5.5	6	6	6
	Ash cohesiveness	6	6	6	6	6
Total		63	68	63.5	62	59
Overall evaluation		Aroma richness improved slightly, accompanied by a subtle fruit note, yet the overall impact remained marginal	The sample exhibited enhanced sweetness and improved cleanness, with a noticeable reduction in off-notes	Maturity showed improvement, while other attributes slightly weakened	Sweetness improved slightly, while off-notes became slightly more pronounced	Light aroma

Further comparison among the different flavonoid groups revealed the following ranking of sensory scores: CCPS group > JPS group > CPG group > HHHT group. Specifically, the cigar tobacco leaves fermented with CCPS exhibited the best sensory quality, characterized by a richer and more abundant aroma, softer and more delicate smoke, sweeter taste, significantly reduced miscellaneous air, and improved palatability.

This improvement may be attributed to the metabolic activities of dominant microorganisms (such as g-Staphylococcus and g-Aspergillus), which decomposed macromolecular proteins and sugars, thereby increasing the total content of organic acids and other volatile flavor compounds in the leaves, ultimately enhancing their sensory quality.

### 3.3 Microbial community analysis

#### 3.3.1 Alpha diversity of bacterial and fungal communities among different fermentation groups

**FIGURE 6:**
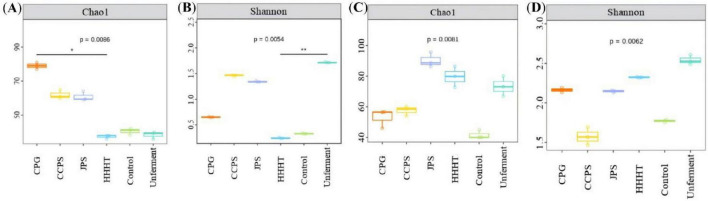
Diversity (Shannon) and relative abundance (Chao1) analysis of bacterial and fungal communities on the surface of cigar tobacco leaves in different fermentation groups. **(A)** Bacterial Chao1 index. **(B)** Bacterial Shannon index. **(C)** Fungal Chao1 index. **(D)** Fungal Shannon index. * represent the significant difference at 0.01 < *p* ≤ 0.05. ** represent the significant difference at 0.001 < *p* ≤ 0.01.

Alpha diversity indices (including Chao1, Good’s coverage, Simpson, Pielou_e, Shannon, and Observed_species) were used to evaluate the richness and diversity of microbial communities within samples (Wu et al., 2023). The ACE and Chao1 indices indicate species richness, whereas the Simpson and Shannon indices reflect community diversity. The Coverage index characterizes the proportion of species covered by sequencing data (Si et al., 2023). As shown in Tables 2, 3 the Good’s coverage values of both bacterial and fungal communities exceeded 0.999, indicating that the sequencing depth was sufficient to cover most species in the samples and that the data were reliable. After 30 days of fermentation, the CPG group exhibited the highest bacterial species richness among the flavonoid-treated groups, followed by the CCPS and JPS groups, whereas the HHHT group showed the lowest bacterial richness (Table 2 and Figures 6A,B). For fungal communities, the JPS group showed the highest species richness, followed by the HHHT and CCPS groups, whereas the CPG group exhibited the lowest fungal richness (Table 3 and Figures 6C,D).

**TABLE 2:** Alpha diversity indices of bacterial communities on the surface of tobacco leaves in different fermentation groups.

Sample	Chao1	Good’s_coverage	Simpson	Pielou_e	Shannon	Observed_species
CPG	79	1	0.193	0.149	0.651	79
CCPS	62	0.99998	0.605	0.354	1.463	62
JPS	60.833	0.99996	0.568	0.328	1.344	60.333
HHHT	37.333	1	0.070	0.067	0.243	37.333
Control	40.5	0.99996	0.106	0.089	0.330	40.333
Unferment	38.417	0.99997	0.769	0.471	1.718	38.333

**TABLE 3:** Alpha diversity indices of fungal communities on tobacco leaf surfaces across different fermentation groups.

Sample	Chao1	Good’s_coverage	Simpson	Pielou_e	Shannon	Observed_species
CPG	53.056	0.9999	0.611	0.379	2.164	52.333
CCPS	57.548	0.9999	0.466	0.372	1.576	55.667
JPS	90.278	0.9998	0.609	0.332	2.146	88.333
HHHT	79.679	0.9998	0.705	0.374	2.324	74.667
Control	41.667	1	0.589	0.329	1.771	41.667
Unferment	73.367	1	0.732	0.411	2.542	73.333

#### 3.3.2 Bacterial and fungal phylum composition and dynamic changes among different fermentation groups

**FIGURE 7:**
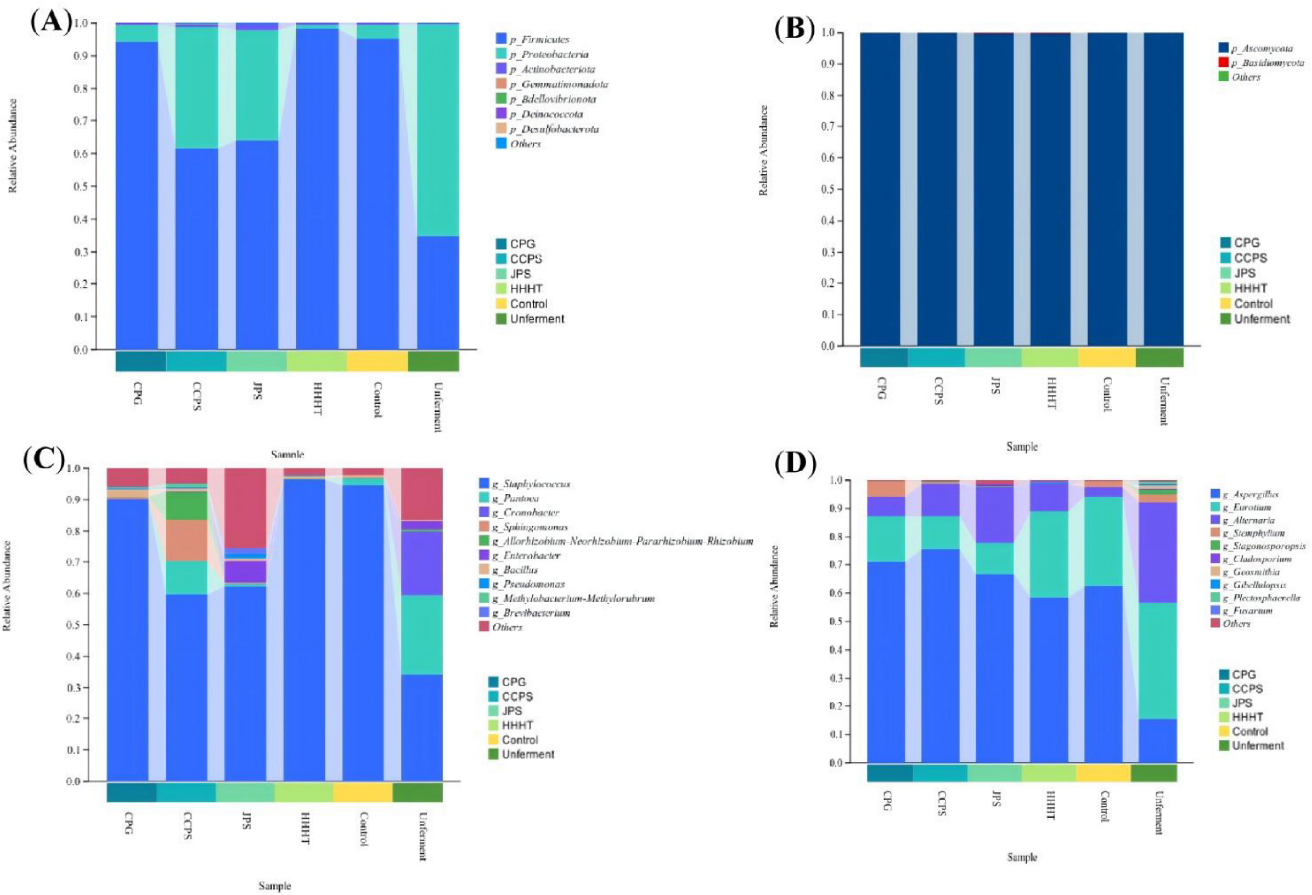
Analysis of the surface bacterial and fungal community composition at the phylum level in cigar tobacco leaves from different flavonoid fermentation groups. **(A)** Bacterial phylum-level composition. **(B)** fungal phylum-level composition. **(C)** Bacterial genus-level composition. **(D)** Fungal genus-level composition.

The phylum-level compositions of bacterial and fungal communities on the surface of tobacco leaves from different fermentation groups are shown in Figures 7A,B. The dominant bacterial phyla were identified as *Firmicutes*, *Proteobacteria*, *Actinobacteriota*, *Gemmatimonadota*, *Bdellovibrionota*, *Deinococcota*, and *Desulfobacterota*; the dominant fungal phylum was *Ascomycota*.

The unfermented group was predominantly composed of *Proteobacteria* (65.2%) and *Firmicutes* (34.6%). After 30 days of fermentation, all treatment groups exhibited a substantial increase in the relative abundance of *Firmicutes* and a decrease in *Proteobacteria*. Among them, the CPG and HHHT groups showed the most dramatic shifts in microbial community structure, with *Firmicutes* becoming the absolute dominant phylum, whereas the changes in the CCPS and JPS groups were relatively moderate. Previous studies have reported that *Actinobacteriota* can influence the chemical composition and sensory quality of tobacco leaves during fermentation through metabolic activities (Wei et al., 2024; Yin et al., 2025; Zhu et al., 2025). Many *Ascomycota* species possess starch-degrading capabilities that contribute to improved tobacco combustion performance and reduced formation of harmful precursor compounds (Yin et al., 2025). In the initial stage of cigar tobacco fermentation, *Proteobacteria* and *Firmicutes* are the dominant bacterial phyla (Si et al., 2023), and the relative abundance of *Firmicutes* increases significantly in the later stages (Zhang et al., 2023). In this study, the changes in the abundance of *Proteobacteria* and *Firmicutes* in the CCPS and JPS groups were consistent with previous reports.

At the genus level (Figures 7C,D), different fermentation treatments with tangerine peel flavonoids significantly altered the community structure of bacteria and fungi on the surface of CTL (*p* < 0.05). For bacteria, all fermentation groups shared a common feature of a marked increase in *Staphylococcus*, with the most significant increase observed in the HHHT group (*p* < 0.05). In contrast, the relative abundances of potentially harmful or spoilage bacteria such as *Pantoea*, *Cronobacter*, and *Enterobacter* decreased significantly (*p* < 0.05). The CCPS group was also accompanied by the enrichment of new dominant genera including *Sphingomonas*, *Rhizobium*, and *Methylobacterium*, resulting in higher microbial diversity. For fungi, compared with the unfermented group, all fermentation groups exhibited a significant increase in *Aspergillus*, obvious decreases in *Eurotium* and *Alternaria*, and a near disappearance of *Septoria* and *Gibberella* (*p* < 0.05). In summary, the fermentation process mediated by tangerine peel flavonoids can directionally enrich core functional microorganisms such as *Staphylococcus* and *Aspergillus*, inhibit potential pathogenic and spoilage microorganisms, reshape the structure and diversity of tobacco leaf microbial communities, and further affect the formation and accumulation of volatile flavor compounds. Previous studies have identified *Staphylococcus* as a core bacterial genus during cigar tobacco fermentation, with its relative abundance increasing during the process (Jia et al., 2023). Previous studies have reported that genera such as *Bacillus* and *Clostridium* are often regarded as potential foodborne pathogens or spoilage bacteria. In this study, a significant reduction in the abundance of these potentially harmful bacteria was observed on the surface of tobacco leaves treated with tangerine peel flavonoids.

#### 3.3.3 Microbial community similarity and differential taxa

**FIGURE 8:**
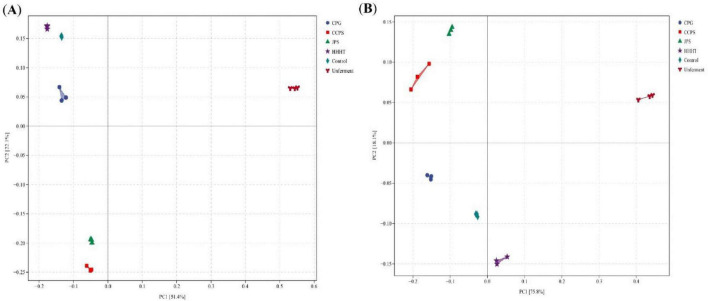
Principal component analysis of bacterial and fungal samples. **(A)** PCoA score plot of bacteria. **(B)** PCoA score plot of fungi.

Principal Coordinate Analysis (PCoA) was performed to assess similarities in microbial community composition among different fermentation groups. In PCoA plots, samples with more similar species compositions clustered closer together. For bacterial communities (Figure 8A), the first two principal coordinates (PC1 and PC2) explained 51.4 and 22.1% of the total variance, respectively. The unfermented sample was distinctly separated from the other samples, indicating marked differences in bacterial community composition. The CCPS and JPS groups, as well as the CPG and HHHT groups, were distributed in different quadrants with considerable distances, indicating distinct surface bacterial community structures among these groups. Samples from different fermentation groups showed clear separation without overlapping. For fungal communities (Figure 8B), the first two principal coordinates (PC1 and PC2) explained 75.8 and 18.1% of the total variance, respectively. The CCPS and JPS groups exhibited smaller distances, indicating similar fungal community compositions. The CPG and control groups were closely clustered, suggesting comparable fungal species compositions. The unfermented sample was clearly separated from the other groups, and the HHHT, CCPS, and JPS groups occupied distinct quadrants with large distances, indicating substantial differences in fungal community composition.

**FIGURE 9:**
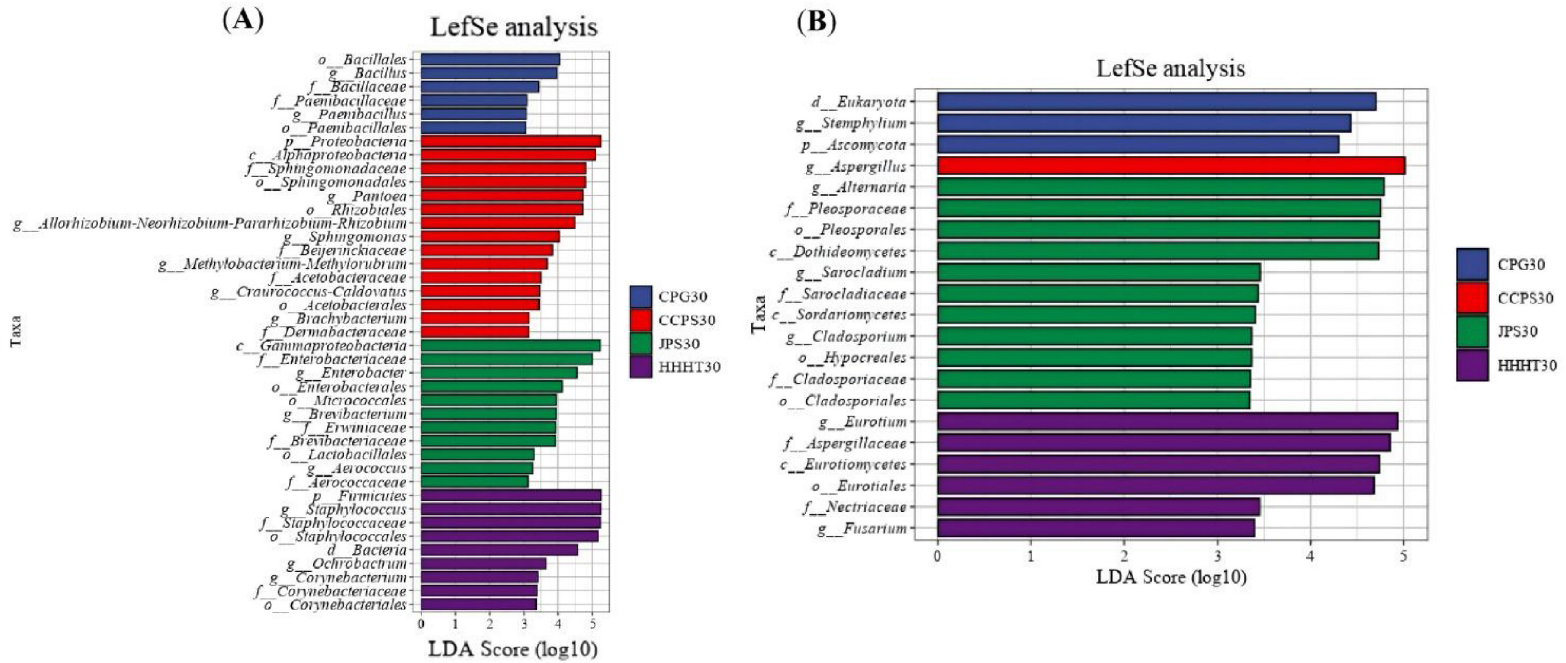
LDA value distribution histogram. **(A)** Bacterial samples. **(B)** Fungal samples.

Linear discriminant analysis effect size (LEfSe) was used to identify microbial biomarkers with statistically significant differences between groups (Linear Discriminant Analysis > 2). As shown in Figure 9A, the CPG group was characterized by seven differential bacterial taxa, including *Bacillus*, *Bacillales*, and *Bacillaceae*. The CCPS group was characterized by 12 differential bacterial taxa, including *Alphaproteobacteria*, *Sphingomonadaceae*, and *Sphingomonadales*. The JPS group was characterized by 16 differential bacterial taxa, including *Enterobacteriaceae* and *Enterobacter*. The HHHT group was characterized by taxa affiliated with *Staphylococcaceae* and *Firmicutes*, with *Staphylococcus* as a dominant genus. The control group showed differential taxa mainly at the phylum level, whereas the unfermented group exhibited seven differential bacterial taxa, including *Gammaproteobacteria* and *Proteobacteria*. The JPS group exhibited the highest number of differential bacterial taxa.

For fungal communities (Figure 9B), the unfermented group was characterized by 15 differential fungal taxa, primarily *Pleosporales*, *Dothideomycetes*, and *Eurotium*. The JPS and control groups were each characterized by five differential fungal taxa, including *Sarocladiaceae*, *Sarocladium*, *Eurotiomycetes*, and *Eurotiales*. The HHHT group was characterized by two differential fungal taxa, namely *Fusarium* and *Nectriaceae*. The CPG and CCPS groups were each characterized by one differential fungal taxon, namely *Stemphylium* and *Aspergillus*, respectively. The unfermented group exhibited the highest number of different fungal taxa.

### 3.4 Correlation between microorganisms and key differential flavor metabolites

**FIGURE 10:**
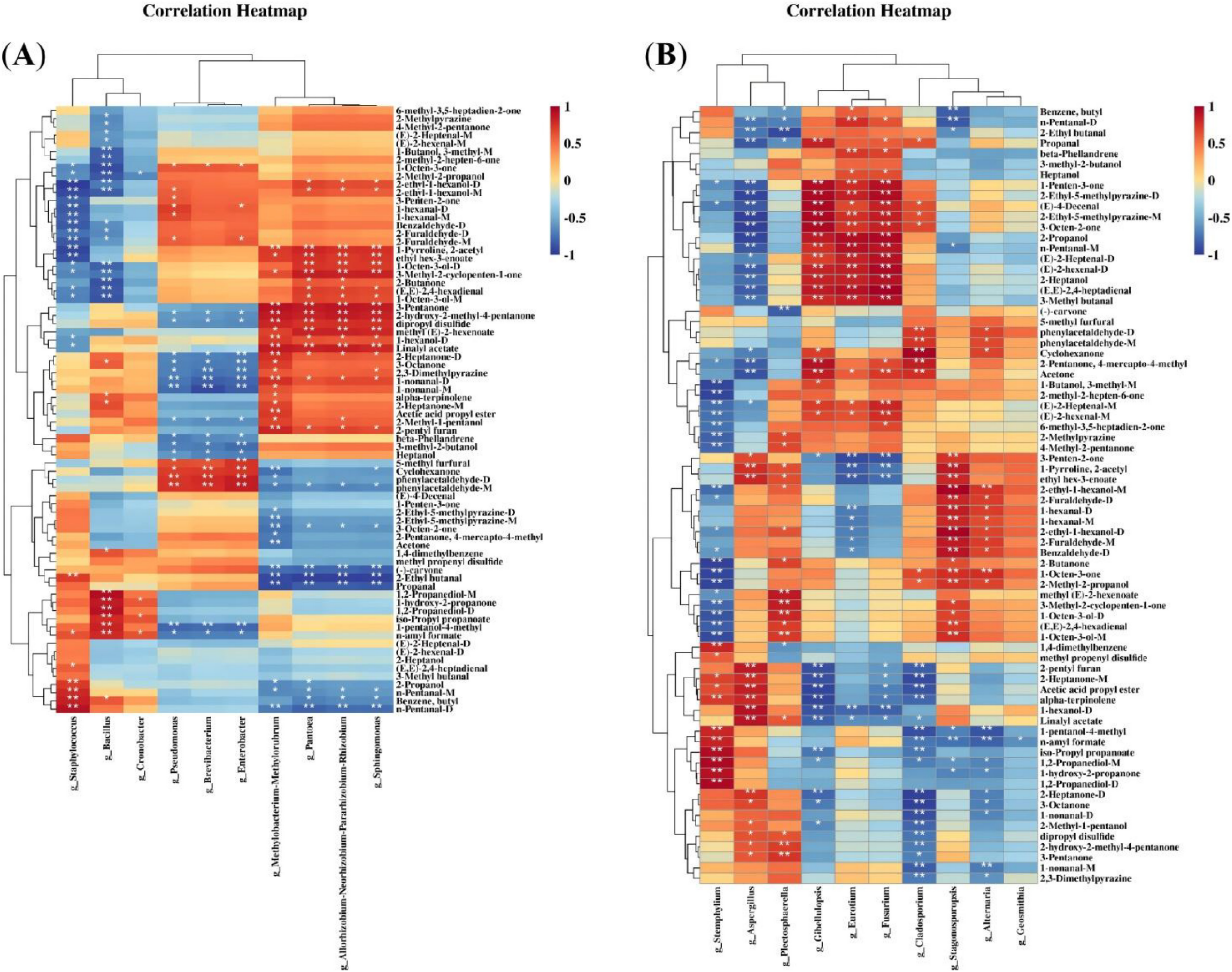
Correlation analysis. **(A)** Bacteria-differential compound correlation heatmap. **(B)** Fungi-differential compound correlation heatmap. * represent the significant difference at 0.01 < *p* ≤ 0.05. ** represent the significant difference at 0.001 < *p* ≤ 0.01.

The formation of volatile compounds in CTL is closely linked to changes in surface microbial communities. Pearson correlation analysis was performed to explore the relationships between the top 10 most abundant microbial genera and differential volatile compounds (Figure 10). As shown in Figures 10A,B, *Staphylococcus* was significantly negatively correlated (*p* < 0.01) with alcohols [e.g., 2-ethyl-1-hexanol-D (*r* = −0.967) and 2-ethyl-1-hexanol-M (*r* = −0.917) and aldehydes (e.g., 1-hexanal-D (*r* = −0.843), 1-hexanal-M (*r* = −0.811), benzaldehyde-D (*r* = −0.853), 2-furaldehyde-D (*r* = −0.755), and 2-furaldehyde-M (*r* = −0.839)], whereas it was significantly positively correlated (*p* < 0.01) with aldehydes such as 2-ethyl butanal (*r* = 0.708), n-pentanal-M (*r* = 0.804), and n-pentanal-D (*r* = 0.913).

*Aspergillus* was significantly positively correlated (*p* < 0.01) with nine volatile compounds, including 2-heptanone-D (*r* = 0.743), 2-pentyl furan (*r* = 0.865), linalyl acetate (*r* = 0.844), ethyl hex-3-enoate (*r* = 0.800) and 1-pyrroline 2-acetyl (*r* = 0.768), whereas it was significantly negatively correlated (*p* < 0.01) with 16 volatile compounds, such as acetone (*r* = −0.870), 2-pentanone, 4-mercapto-4-methyl (*r* = −0.838), 1-penten-3-one (*r* = −0.844), 3-methyl butanal (*r* = −0.745), n-pentanal-D (*r* = −0.720), and propanal (*r* = −0.845).

## 4 Discussion

The flavor quality of tobacco leaves is directly associated with the types and concentrations of their volatile components. In this study, the total volatile content of cigar tobacco leaves (CTL) fermented with tangerine peel flavonoids was significantly lower than that of the unfermented group, a phenomenon that may be attributed to microbial metabolism during fermentation. Specifically, certain volatile components are transformed into smaller molecules during this process, which in turn leads to a reduction in the overall content of flavor-related volatile compounds (Zhang et al., 2025). Further principal component analysis (PCA) and orthogonal partial least squares discriminant analysis (OPLS-DA) revealed significant differences in volatile components between the flavonoid-treated groups and both the control and unfermented groups, as well as among the flavonoid-treated groups themselves. Notably, each flavonoid-treated group exhibited distinct characteristic flavor profiles: the CPG group was characterized by the lowest contents of alcohols and ketones but the highest contents of esters and acids (e.g., n-amyl formate and propyl acetate), which are closely associated with fruity aromas. In contrast, the CCPS group showed high levels of ketones (e.g., 3-methyl-2-cyclopenten-1-one, 3-pentanone, and 2-hydroxy-2-methyl-4-pentanone), which contribute to a caramel-like sweetness and enhanced smoke body. These differences in volatile profiles are closely associated with changes in the surface microbial communities induced by different tangerine peel flavonoid media. Microbial analysis showed that the CPG group had the highest bacterial richness, the JPS group had the highest fungal richness, and the HHHT group had the lowest overall microbial diversity. Among the bacterial communities, a significant increase in *Firmicutes* and a decrease in *Proteobacteria* were observed, with *Staphylococcus* emerging as the dominant genus—consistent with the findings of previous studies (Zhang et al., 2025). In addition, the abundance of potentially harmful bacteria (e.g., Pantoea and Cronobacter) was reduced. The fungal community, meanwhile, was dominated by *Ascomycota*, with a marked increase in *Aspergillus* and a decrease in *Scopulariopsis* and *Cladosporium*. Correlation analysis further confirmed significant associations between microorganisms and flavor compounds: for example, *Staphylococcus* showed negative correlations with certain aldehydes and esters, whereas Aspergillus exhibited significant positive correlations with various aroma-producing compounds.

The underlying mechanism for this phenomenon may be that the exogenous addition of three tangerine peel flavonoids (CPG, CCPS, and JPS) can affect the synthesis and accumulation of volatile compounds by regulating the microbial community structure and core metabolic pathways during CTL fermentation. Specifically, these flavonoids mainly improve the relative abundances of *Staphylococcus* and *Aspergillus* at the end of fermentation, thereby further influencing the flavor quality of CTL.

*Staphylococcus*, as a major bacterial community in CTL fermentation, is involved in fatty acid and amino acid metabolism, and its abundance is closely related to the formation of volatile compounds (Zhang et al., 2025). When CPG, CCPS, and JPS are exogenously added, they may competitively bind to key enzymes in fatty acid synthesis and decomposition, thereby inhibiting the β-oxidation of fatty acids, the carbon chain elongation of long-chain fatty acids, and their conversion to aldehydes and alcohols (Zong-Tsi et al., 2012); this ultimately reduces the synthesis of long-chain fatty alcohols and long-chain aldehydes. Meanwhile, these flavonoids may downregulate the expression of transaminase, decarboxylase, aldehyde dehydrogenase, and other related enzymes, which could inhibit the decomposition of aromatic amino acids and heterocyclic compounds (Xue et al., 2023) and may reduce the production of heterocyclic aldehydes such as benzofuran one and 2-furanone. They also inhibit the activities of alcohol dehydrogenase and aldehyde reductase, blocking the conversion pathway from fatty acids/amino acids to aldehydes and then to alcohols (Cai et al., 2024). Consequently, the abundance of *Staphylococcus* is negatively correlated with the contents of the aforementioned substances.

*Aspergillus*, a major fungal community in fermentation, plays an important role in the formation of flavor compounds due to its strong secondary metabolic capacity, which enables it to degrade or synthesize a variety of volatile flavor compounds (Haiqing et al., 2024; Zhong et al., 2024). On the one hand, its metabolism consumes large amounts of precursor substances such as fatty acids, terpenoids, and amino acids; meanwhile, *Aspergillus* secretes esterases, ketone-degrading enzymes, and heterocyclic compound-degrading enzymes, which can degrade substances such as 2-heptanone, linalyl acetate, and 1-pyrroline, leading to a decrease in their contents. Tangerine peel flavonoids may act synergistically with the metabolic enzyme system of *Aspergillus* to enhance the inhibition and degradation of the above substances, resulting in a negative correlation between Aspergillus abundance and the contents of these compounds (Zhong et al., 2024; Daniel et al., 2024). On the other hand, *Aspergillus* can directly synthesize a variety of volatile compounds through pathways such as sugar metabolism, lipid metabolism, amino acid decarboxylation, and sulfur-containing amino acid decomposition, including 16 volatile substances such as acetone, 2-pentanone, 3-methylbutanal, propanal, and 4-mercapto-4-methyl-1-penten-3-one. During this process, flavonoids may upregulate the expression of key enzymes in *Aspergillus*, such as acetyl-CoA synthase, ketone body synthase, and sulfur-containing amino acid-degrading enzymes, thereby promoting the supply of precursor substances and improving metabolic efficiency. Consequently, *Aspergillus* abundance shows an extremely significant positive correlation with the contents of such volatile compounds (Daniel et al., 2024)

## 5 Conclusion

A clear understanding of the effects of tangerine peel flavonoids on cigar tobacco fermentation is important for optimizing cigar flavor and quality. In this study, the effects of different tangerine peel flavonoid treatments on the flavor components and microbial communities of CTL were systematically investigated using GC-IMS, 16S rRNA gene sequencing, and ITS sequencing. The results demonstrated that the CCPS fermentation group was characterized by flavor compounds such as 3-methyl-2-cyclopenten-1-one, 3-pentanone, and 2-hydroxy-2-methyl-4-pentanone, whereas n-amyl formate and propyl acetate were enriched in the CPG group. Several bacterial genera (e.g., *Staphylococcus* and *Pantoea*) and fungal genera (e.g., *Aspergillus*, *Eurotium*, and *Alternaria*) were identified as the main microorganisms involved in fermentation, with significant correlations observed between these genera and key differential flavor compounds. This study provides theoretical support and practical guidance for improving the quality and flavor of CTL through targeted fermentation with tangerine peel flavonoids.

## Data Availability

The data is deposited in NCBI with accession numbers: PRJNA1453136 and PRJNA1453131.

## Footnotes

1
https://www.genescloud.cn/
