# Supplementary material for: Effects of tangerine peel flavonoids on flavor compounds and microbial communities in cigar tobacco fermentation: insights from ion migration chromatography and microbiomics
Source: Front Microbiol. 2026 May 4;17:1793725. doi: 10.3389/fmicb.2026.1793725 (PMC13180802; doi:10.3389/fmicb.2026.1793725)
Supplement: Supplementary file 1 [file Table_1.docx]

Supplementary Material

# Supplementary Tables

Table S1. Differential Compounds in the CPG, CCPS, JPS, HHHT, Control and Unfermented Groups

| **Class** | **Compound** | **VIP** |  | **Content(mg/kg)** | | | | | |
| --- | --- | --- | --- | --- | --- | --- | --- | --- | --- |
|  |  |  | **CAS** | **CPG** | **CCPS** | **JPS** | **HHHT** | **Control** | **Unferment** |
| Ketones | (-)-carvone | 1.33941 | C99490 | 0.063±0.002 | 0.051±0.003 | 0.064±0.001 | 0.058±0.002 | 0.055±0.001 | 0.055±0.003 |
|  | 1-Octen-3-one | 1.21483 | C4312996 | 0.147±0.009 | 0.227±0.003 | 0.248±0.006 | 0.213±0.013 | 0.186±0.005 | 0.169±0.012 |
|  | Cyclohexanone | 1.19332 | C108941 | 0.088±0.004 | 0.085±0.006 | 0.160±0.002 | 0.137±0.014 | 0.080±0.001 | 0.102±0.002 |
|  | 2-Heptanone-M | 1.16479 | C110430 | 0.873±0.025 | 0.793±0.029 | 0.389±0.034 | 0.390±0.012 | 0.624±0.009 | 0.531±0.009 |
|  | 1-hydroxy-2-propanone | 1.12095 | C116096 | 0.135±0.018 | 0.089±0.001 | 0.083±0.003 | 0.092±0.007 | 0.110±0.007 | 0.079±0.002 |
|  | 2-Octanone-M | 1.07934 | C111137 | 0.644±0.031 | 0.757±0.057 | 0.749±0.009 | 0.694±0.031 | 0.854±0.013 | 0.732±0.014 |
|  | 2-Nonanone | 1.04622 | C821556 | 0.076±0.001 | 0.084±0.002 | 0.080±0.001 | 0.082±0.004 | 0.089±0.002 | 0.084±0.002 |
|  | 2-Heptanone-D | 1.03569 | C110430 | 0.136±0.002 | 0.141±0.013 | 0.071±0.009 | 0.089±0.003 | 0.294±0.014 | 0.121±0.002 |
|  | 3-Octanone | 1.00784 | C106683 | 0.118±0.006 | 0.118±0.003 | 0.097±0.002 | 0.103±0.004 | 0.242±0.017 | 0.105±0.004 |
| Aldehydes | (E,E)-2,4-heptadienal | 1.33485 | C4313035 | 0.205±0.002 | 0.211±0.005 | 0.213±0.003 | 0.265±0.006 | 0.242±0.003 | 0.232±0.010 |
|  | 1-nonanal-D | 1.25234 | C124196 | 0.195±0.010 | 0.213±0.006 | 0.147±0.015 | 0.178±0.008 | 0.140±0.005 | 0.177±0.018 |
|  | 1-nonanal-M | 1.2287 | C124196 | 0.718±0.019 | 0.740±0.005 | 0.651±0.030 | 0.709±0.010 | 0.636±0.005 | 0.691±0.033 |
|  | 5-methyl furfural | 1.14286 | C620020 | 0.343±0.020 | 0.326±0.006 | 0.366±0.017 | 0.324±0.013 | 0.319±0.014 | 0.360±0.019 |
|  | n-Pentanal-M | 1.11769 | C110623 | 0.574±0.015 | 0.527±0.006 | 0.355±0.003 | 0.657±0.037 | 0.650±0.130 | 0.454±0.010 |
|  | Propanal | 1.08454 | C123386 | 0.590±0.005 | 0.541±0.007 | 0.619±0.009 | 0.636±0.030 | 0.552±0.018 | 0.677±0.017 |
|  | (Z)-2-octenal | 1.05729 | C20664464 | 0.104±0.008 | 0.077±0.011 | 0.079±0.001 | 0.075±0.001 | 0.097±0.007 | 0.074±0.007 |
|  | 2-Ethyl butanal | 1.03914 | C97961 | 0.165±0.006 | 0.129±0.003 | 0.161±0.002 | 0.168±0.008 | 0.177±0.000 | 0.219±0.014 |
|  | n-Pentanal-D | 1.0338 | C110623 | 1.422±0.046 | 1.208±0.020 | 1.507±0.021 | 1.473±0.058 | 1.030±0.010 | 1.670±0.044 |
|  | 3-Methyl butanal | 1.01748 | C590863 | 1.531±0.007 | 1.560±0.059 | 1.553±0.013 | 1.691±0.026 | 1.372±0.023 | 1.761±0.018 |
|  | (E,E)-2,4-hexadienal | 1.00665 | C142836 | 0.066±0.002 | 0.081±0.002 | 0.076±0.002 | 0.074±0.003 | 0.066±0.002 | 0.097±0.002 |
|  | 1-hexanal-M | 1.00637 | C66251 | 0.512±0.081 | 0.611±0.005 | 0.633±0.003 | 0.489±0.073 | 0.683±0.046 | 0.874±0.039 |
| Alcohols | Butanol-D | 1.21985 | C71363 | 0.813±0.005 | 0.835±0.023 | 0.830±0.020 | 0.909±0.036 | 1.814±0.004 | 0.783±0.001 |
|  | Butanol-M | 1.15275 | C71363 | 1.499±0.018 | 1.484±0.032 | 1.436±0.033 | 1.508±0.060 | 0.410±0.011 | 1.432±0.007 |
|  | 1-pentanol-4-methyl | 1.13616 | C626891 | 0.165±0.006 | 0.145±0.002 | 0.123±0.002 | 0.142±0.004 | 0.156±0.003 | 0.168±0.001 |
|  | 1,2-Propanediol-D | 1.12127 | C57556 | 0.083±0.028 | nd | nd | nd | nd | nd |
|  | 1-Butanol, 3-methyl-M | 1.08488 | C123513 | 1.049±0.011 | 1.098±0.001 | 1.097±0.003 | 1.112±0.009 | 1.112±0.002 | 1.099±0.006 |
|  | 3-methyl-2-butanol | 1.07908 | C598754 | 0.105±0.004 | 0.120±0.001 | 0.096±0.004 | 0.122±0.004 | 0.126±0.007 | 0.072±0.002 |
|  | 1,2-Propanediol-M | 1.06907 | C57556 | 0.561±0.088 | 0.202±0.007 | 0.111±0.007 | 0.159±0.007 | 0.077±0.004 | 0.086±0.007 |
|  | 2-Methyl-1-pentanol | 1.03279 | C105306 | 0.122±0.006 | 0.124±0.002 | 0.113±0.001 | 0.117±0.003 | 0.109±0.002 | 0.131±0.004 |
|  | 2-hexanol | 1.02684 | C626937 | 0.393±0.017 | 0.428±0.016 | 0.457±0.018 | 0.455±0.008 | 0.380±0.006 | 0.375±0.005 |
|  | 1-Octen-3-ol-D | 1.02361 | C3391864 | 0.103±0.001 | 0.219±0.002 | 0.171±0.005 | 0.173±0.004 | 0.143±0.004 | nd |
|  | 2-Methyl-2-propanol | 1.01819 | C67630 | 1.325±0.008 | 1.364±0.006 | 1.367±0.003 | 1.356±0.005 | 1.121±0.023 | 1.308±0.014 |
|  | 3-Methyl-3-buten-1-ol | 1.01458 | C763326 | 0.216±0.005 | 0.216±0.001 | 0.233±0.001 | 0.223±0.012 | 0.274±0.008 | 0.216±0.006 |
|  | 2-Pentanol-M | 1.00413 | C6032297 | 0.898±0.009 | 0.933±0.010 | 0.936±0.016 | 0.981±0.016 | 0.830±0.005 | 0.996±0.019 |
| Lipids | n-amyl formate | 1.31054 | C638493 | 0.330±0.019 | 0.274±0.016 | 0.241±0.002 | 0.282±0.014 | 0.291±0.004 | 0.279±0.003 |
|  | methyl (E)-2-hexenoate | 1.15174 | C13894638 | 0.141±0.004 | 0.163±0.003 | 0.148±0.002 | 0.151±0.007 | 0.127±0.006 | 0.145±0.006 |
|  | ethyl hex-3-enoate | 1.12551 | C2396830 | 0.266±0.007 | 0.291±0.005 | 0.278±0.008 | 0.252±0.007 | 0.297±0.000 | 0.242±0.005 |
|  | Acetic acid propyl ester | 1.10698 | C109604 | 0.502±0.014 | 0.477±0.002 | 0.210±0.003 | 0.162±0.006 | 0.044±0.005 | 0.261±0.007 |
|  | propyl thioacetate | 1.05376 | C2307100 | 0.281±0.011 | 0.244±0.019 | 0.211±0.010 | 0.219±0.009 | 0.261±0.008 | 0.291±0.009 |
|  | iso-Propyl propanoate | 1.02515 | C637785 | 0.172±0.015 | 0.123±0.009 | 0.101±0.003 | 0.098±0.005 | 0.080±0.008 | 0.109±0.005 |
| Alkenes | alpha-terpinolene | 1.06052 | C586629 | 0.301±0.009 | 0.282±0.005 | 0.241±0.005 | 0.225±0.004 | 0.286±0.005 | 0.316±0.025 |
|  | beta-Phellandrene | 1.05924 | C555102 | 0.321±0.011 | 0.324±0.001 | 0.310±0.003 | 0.339±0.010 | 0.260±0.004 | 0.319±0.002 |
| Others | methyl propenyl disulfide | 1.14761 | C5905475 | 0.090±0.000 | 0.082±0.001 | 0.091±0.001 | 0.083±0.006 | 0.088±0.005 | 0.100±0.002 |
|  | 2,3-dimethyl-5-ethylpyrazine | 1.13498 | C15707343 | 1.314±0.007 | 0.320±0.005 | 0.330±0.003 | 0.341±0.003 | 0.334±0.006 | 0.324±0.007 |
|  | 2-pentyl furan | 1.12917 | C3777693 | 0.300±0.006 | 0.310±0.014 | 0.259±0.002 | 0.256±0.007 | 0.308±0.007 | 0.320±0.015 |
|  | Benzene, butyl | 1.09518 | C104518 | 0.183±0.008 | 0.168±0.001 | 0.132±0.001 | 0.182±0.002 | 0.153±0.002 | 0.173±0.002 |

Table S2. Differential Compounds in the CPG, CCPS, JPS and HHHT Groups

| **Class** | **Compound** | **VIP** | **CAS** | **Content(mg/kg)** | | | |
| --- | --- | --- | --- | --- | --- | --- | --- |
|  |  |  |  | **CPG** | **CCPS** | **JPS** | **HHHT** |
| Aldehydes | phenylacetaldehyde-M | 1.17986 | C122781 | 0.065±0.014 | 0.625±0.004 | 0.717±0.016 | 0.654±0.017 |
|  | phenylacetaldehyde-D | 1.15088 | C122781 | 0.662±0.002 | 0.056±0.003 | 0.073±0.004 | 0.060±0.005 |
|  | 2-Ethyl butanal | 1.12141 | C97961 | 0.165±0.006 | 0.129±0.003 | 0.161±0.002 | 0.168±0.008 |
|  | n-Pentanal-D | 1.11907 | C110623 | 1.422±0.046 | 1.208±0.020 | 1.507±0.021 | 1.473±0.058 |
|  | 1-nonanal-D | 1.1103 | C124196 | 0.195±0.010 | 0.213±0.006 | 0.147±0.015 | 0.178±0.008 |
|  | (E)-2-Heptenal-D | 1.09764 | C18829555 | 0.047±0.002 | 0.056±0.001 | 0.049±0.004 | 0.071±0.002 |
|  | 1-nonanal-M | 1.09489 | C124196 | 0.718±0.019 | 0.740±0.005 | 0.651±0.030 | 0.709±0.010 |
|  | n-Pentanal-M | 1.08914 | C110623 | 0.574±0.015 | 0.527±0.006 | 0.355±0.003 | 0.657±0.037 |
|  | Benzaldehyde-D | 1.08311 | C100527 | 1.461±0.040 | 1.557±0.019 | 1.850±0.032 | 1.475±0.025 |
|  | (E,E)-2,4-heptadienal | 1.08146 | C4313035 | 0.205±0.002 | 0.211±0.005 | 0.213±0.003 | 0.265±0.006 |
|  | Propanal | 1.07301 | C123386 | 0.590±0.005 | 0.541±0.007 | 0.619±0.009 | 0.636±0.030 |
|  | (E,E)-2,4-hexadienal | 1.06021 | C142836 | 0.066±0.002 | 0.081±0.002 | 0.076±0.002 | 0.074±0.003 |
|  | 1-hexanal-D | 1.05788 | C66251 | 0.059±0.021 | 0.090±0.003 | 0.097±0.005 | 0.052±0.018 |
|  | 2-Furaldehyde-M | 1.05447 | C98011 | 0.596±0.020 | 0.639±0.010 | 0.648±0.018 | 0.598±0.017 |
|  | 5-methyl furfural | 1.05264 | C620020 | 0.343±0.020 | 0.326±0.006 | 0.366±0.017 | 0.324±0.013 |
|  | (E)-2-hexenal-D | 1.05075 | C6728263 | 1.471±0.005 | 0.498±0.023 | 0.503±0.012 | 0.598±0.025 |
|  | (E)-2-Heptenal-M | 1.03557 | C18829555 | 0.288±0.009 | 0.344±0.005 | 0.326±0.011 | 0.385±0.013 |
|  | (E)-2-hexenal-M | 1.02321 | C6728263 | 1.041±0.016 | 1.087±0.007 | 1.071±0.002 | 1.117±0.018 |
|  | 3-Methyl butanal | 1.01968 | C590863 | 1.531±0.007 | 1.560±0.059 | 1.553±0.013 | 1.691±0.026 |
|  | 1-hexanal-M | 1.01931 | C66251 | 0.512±0.081 | 0.611±0.005 | 0.633±0.003 | 0.489±0.073 |
|  | (E)-4-Decenal | 1.01292 | C65405701 | 0.050±0.002 | 0.055±0.001 | 0.063±0.002 | 0.072±0.002 |
|  | 2-Furaldehyde-D | 1.01242 | C98011 | 0.095±0.008 | 0.111±0.000 | 0.114±0.006 | 0.100±0.008 |
| Alcohols | 2-ethyl-1-hexanol-D | 1.17112 | C104767 | 0.286±0.001 | 0.340±0.001 | 0.333±0.000 | 0.292±0.004 |
|  | 2-ethyl-1-hexanol-M | 1.15447 | C104767 | 0.889±0.002 | 0.953±0.003 | 0.949±0.002 | 0.909±0.002 |
|  | Heptanol | 1.13728 | C53535334 | 0.167±0.004 | 0.174±0.001 | 0.163±0.001 | 0.177±0.004 |
|  | 3-methyl-2-butanol | 1.13276 | C598754 | 0.105±0.004 | 0.120±0.001 | 0.096±0.004 | 0.122±0.004 |
|  | 1-Octen-3-ol-D | 1.10669 | C3391864 | 0.103±0.001 | 0.219±0.002 | 0.171±0.005 | 0.173±0.004 |
|  | 1-Octen-3-ol-M | 1.10431 | C3391864 | 1.143±0.016 | 1.500±0.006 | 1.397±0.005 | 0.356±0.005 |
|  | 2-Propanol | 1.10063 | C67630 | 1.237±0.047 | 1.156±0.006 | 1.259±0.034 | 1.553±0.023 |
|  | 1-pentanol-4-methyl | 1.09069 | C626891 | 0.165±0.006 | 0.145±0.002 | 0.123±0.002 | 0.142±0.004 |
|  | 2-Methyl-2-propanol | 1.04377 | C67630 | 1.325±0.008 | 1.364±0.006 | 1.367±0.003 | 1.356±0.005 |
|  | 2-Heptanol | 1.04323 | C543497 | 0.134±0.007 | 0.142±0.004 | 1.142±0.005 | 0.166±0.001 |
|  | 1,2-Propanediol-M | 1.02755 | C57556 | 0.561±0.088 | 0.202±0.007 | 0.111±0.007 | 0.159±0.007 |
|  | 2-Methyl-1-pentanol | 1.0113 | C105306 | 0.122±0.006 | 0.124±0.002 | 0.113±0.001 | 0.117±0.003 |
|  | 1,2-Propanediol-D | 1.00633 | C57556 | 0.083±0.028 | nd | nd | nd |
|  | 1-Butanol, 3-methyl-M | 1.00229 | C123513 | 1.049±0.011 | 1.098±0.001 | 1.097±0.003 | 1.112±0.009 |
|  | 1-hexanol-D | 1.00202 | C111273 | 0.292±0.005 | 0.298±0.002 | 0.286±0.002 | 0.273±0.007 |
| Ketones | (-)-carvone | 1.12981 | C99490 | 0.063±0.002 | 0.051±0.003 | 0.064±0.001 | 0.058±0.002 |
|  | 3-Methyl-2-cyclopenten-1-one | 1.12913 | C2758181 | 0.181±0.003 | 0.278±0.002 | 0.229±0.001 | 0.226±0.003 |
|  | 3-Pentanone | 1.09556 | C96220 | 0.467±0.013 | 0.500±0.007 | 0.450±0.005 | 0.462±0.005 |
|  | 3-Penten-2-one | 1.09403 | C625332 | 0.044±0.003 | 0.047±0.000 | 0.048±0.002 | 0.037±0.002 |
|  | 2-Heptanone-D | 1.08995 | C110430 | 0.136±0.002 | 0.141±0.013 | 0.071±0.009 | 0.089±0.003 |
|  | 2-hydroxy-2-methyl-4-pentanone | 1.08956 | C123422 | 0.235±0.004 | 0.255±0.009 | 0.227±0.001 | 0.235±0.004 |
|  | 4-Methyl-2-pentanone | 1.08725 | C108101 | 2.167±0.003 | 0.210±0.004 | 0.183±0.001 | 0.209±0.005 |
|  | 1-Octen-3-one | 1.08561 | C4312996 | 0.147±0.009 | 0.227±0.003 | 0.248±0.006 | 0.213±0.013 |
|  | Cyclohexanone | 1.08011 | C108941 | 0.088±0.004 | 0.085±0.006 | 0.160±0.002 | 0.137±0.014 |
|  | 2-Butanone | 1.07272 | C78933 | 4.444±0.005 | 2.653±0.006 | 2.593±0.005 | 2.603±0.011 |
|  | 6-methyl-3,5-heptadien-2-one | 1.05224 | C1604280 | 0.063±0.001 | 0.079±0.004 | 0.069±0.001 | 0.083±0.002 |
|  | 3-Octanone | 1.0473 | C106683 | 0.118±0.006 | 0.118±0.003 | 0.097±0.002 | 0.103±0.004 |
|  | 3-Octen-2-one | 1.04597 | C1669449 | 0.514±0.027 | 0.499±0.005 | 0.598±0.010 | 0.675±0.017 |
|  | 2-Heptanone-M | 1.04957 | C110430 | 0.873±0.025 | 0.793±0.029 | 0.389±0.034 | 0.390±0.012 |
|  | 2-Pentanone, 4-mercapto-4-methyl | 1.03755 | C19872527 | 0.308±0.004 | 0.312±0.001 | 0.348±0.007 | 0.354±0.003 |
|  | 2-methyl-2-hepten-6-one | 1.02881 | C110930 | 3.406±0.073 | 3.719±0.007 | 3.649±0.002 | 3.727±0.027 |
|  | 1-Penten-3-one | 1.02493 | C1629589 | 0.420±0.007 | 0.447±0.012 | 0.464±0.013 | 0.537±0.014 |
|  | Acetone | 1.0065 | C67641 | 4.932±0.010 | 4.932±0.009 | 4.972±0.008 | 4.987±0.013 |
|  | 1-hydroxy-2-propanone | 1.00432 | C116096 | 0.135±0.018 | 0.089±0.001 | 0.083±0.003 | 0.092±0.007 |
| Lipids | ethyl hex-3-enoate | 1.13002 | C2396830 | 0.266±0.007 | 0.291±0.005 | 0.278±0.008 | 0.252±0.007 |
|  | n-amyl formate | 1.09174 | C638493 | 0.330±0.019 | 0.274±0.016 | 0.241±0.002 | 0.282±0.014 |
|  | Acetic acid propyl ester | 1.04914 | C109604 | 0.502±0.014 | 0.477±0.002 | 0.210±0.003 | 0.162±0.006 |
|  | Linalyl acetate | 1.04476 | C115957 | 0.289±0.003 | 0.292±0.004 | 0.278±0.004 | 0.273±0.003 |
|  | methyl (E)-2-hexenoate | 1.0434 | C13894638 | 0.141±0.004 | 0.163±0.003 | 0.148±0.002 | 0.151±0.007 |
|  | iso-Propyl propanoate | 1.0031 | C637785 | 0.172±0.015 | 0.123±0.009 | 0.101±0.003 | 0.098±0.005 |
| Alkenes | alpha-terpinolene | 1.02141 | C586629 | 0.301±0.009 | 0.282±0.005 | 0.241±0.005 | 0.225±0.004 |
|  | beta-Phellandrene | 1.0212 | C555102 | 0.321±0.011 | 0.324±0.001 | 0.310±0.003 | 0.339±0.010 |
| Others | 1-Pyrroline, 2-acetyl | 1.15699 | C85213225 | 0.123±0.002 | 0.138±0.003 | 0.131±0.001 | 0.117±0.002 |
|  | 2-Ethyl-5-methylpyrazine-D | 1.05542 | C13360640 | 0.077±0.003 | 0.081±0.003 | 0.098±0.004 | 0.134±0.002 |
|  | 2-pentyl furan | 1.05214 | C3777693 | 0.300±0.006 | 0.310±0.014 | 0.259±0.002 | 0.256±0.007 |
|  | 2-Ethyl-5-methylpyrazine-M | 1.04574 | C13360640 | 0.382±0.025 | 0.371±0.004 | 0.478±0.013 | 0.576±0.019 |
|  | methyl propenyl disulfide | 1.0401 | C5905475 | 0.090±0.000 | 0.082±0.001 | 0.091±0.001 | 0.083±0.006 |
|  | 1,4-dimethylbenzene | 1.03149 | C106423 | 0.561±0.021 | 0.452±0.016 | 0.509±0.015 | 0.448±0.021 |
|  | 2-Methylpyrazine | 1.03074 | C109080 | 0.175±0.004 | 0.222±0.001 | 0.197±0.004 | 0.223±0.017 |
|  | dipropyl disulfide | 1.02681 | C629196 | 0.369±0.011 | 0.390±0.001 | 0.346±0.003 | 0.359±0.016 |
|  | 2,3-Dimethylpyrazine | 1.02062 | C5910894 | 0.042±0.002 | 0.042±0.002 | 0.037±0.001 | 0.040±0.001 |
|  | Benzene, butyl | 1.00737 | C104518 | 0.183±0.008 | 0.168±0.001 | 0.132±0.001 | 0.182±0.002 |
